# Effect of antisecretory factor, given as a food supplement to adult patients with severe traumatic brain injury (SASAT): protocol for an exploratory randomized double blind placebo-controlled trial

**DOI:** 10.1186/s13063-022-06275-z

**Published:** 2022-04-23

**Authors:** David Cederberg, Bradley M. Harrington, Adriaan Johannes Vlok, Peter Siesjö

**Affiliations:** 1 Department of Neurosurgery, Skane University Hospital, Lund, Sweden; 2 Department of Neurosurgery, Tygerberg University Hospital, Tygerberg, Cape Town, Republic of South Africa

## Abstract

**Background:**

Traumatic brain injury (TBI) constitutes a global epidemic. Overall outcome is poor, with mortality ranging from 10 to 70% and significant long-term morbidity. Several experimental reports have claimed effect on traumatic edema, but no clinical trials have shown effect on edema or outcome. Antisecretory factor, an endogenous protein, is commercially available as Salovum®, which is classified as a medical food by the European Union and has shown effect in experimental trauma models and feasibility with signs of effect in 2 pilot case series. The aim of this study is to assess the effect of antisecretory factor in adult patients with severe traumatic brain injury as measured by 30-day mortality, treatment intensity level (TIL), and intracranial pressure (ICP).

**Methods/design:**

This is a single-center, double-blind, randomized, placebo-controlled clinical phase 2 trial, investigating the clinical superiority of Salovum® given as a food supplement to adults with severe TBI (GCS < 9), presenting to the trauma unit at Tygerberg University Hospital, Cape Town, South Africa, that are planned for invasive ICP monitoring and neurointensive care, will be screened for eligibility, and assigned to either treatment group (*n* = 50) or placebo group (*n* = 50). In both groups, the primary outcome will be 30-day mortality, recorded via hospital charts, follow-up phone calls, and the population registry. Secondary outcomes will be treatment intensity level (TIL), scored from hospital charts, and ICP registered from hospital data monitoring.

**Trial registration:**

ClinicalTrials.gov NCT03339505. Registered on September 17, 2017.

Protocol version 3.0 from November 13, 2020

**Supplementary Information:**

The online version contains supplementary material available at 10.1186/s13063-022-06275-z.

## Introduction

### Background and rationale

Traumatic brain injury (TBI) constitutes a global burden despite the fact that mortality and morbidity have been reduced in several countries during the last decades [11, 13]. Advances in neurointensive care, cerebral monitoring, and neuroradiology have improved outcome for patients with severe TBI, but the results globally are still poor, with a mortality ranging from 10 to 70% and significant long-term morbidity [18].

Traumatic brain injury encompasses several pathogenic mechanisms such as primary mechanical injury and hemorrhage followed by secondary events such as vasospasm, inflammation, excitotoxic cell damage, and energy deprivation, but also long-term progressive brain tissue degeneration. One common denominator in TBI is cerebral edema, which may cause raised intracranial pressure (ICP) and is a major factor responsible for mortality and morbidity in TBI [21]. The pathophysiologic mechanisms of cerebral edema are, however, only partially known [20].

Although several experimental reports have claimed effect on traumatic cerebral edema, all clinical trials have failed [2].

Antisecretory factor (AF) is a 41-kDa endogenous protein proposed to possess both antisecretory and anti-inflammatory effects [16]. The exact mechanism of AF is unknown, but it has been proposed to act by modulation of proteasomes, complement, and myeloid cells [5, 14, 22]. A recent report shows that AF inhibits the NKCC1 ion pump which also has been implicated in the evolution of edema in TBI [10, 23].

Salovum® is an egg yolk powder enriched for AF and classified as food for specific medical purposes in the EU. Salovum has been used in clinical trials for gastroenteritis, Meniere disease, and inflammatory bowel disease with no toxicity reported [15]. Salovum is currently registered in the Republic of South Africa.

An active part of AF has been synthesized within a 16-amino-acid peptide, AF-16. AF-16 and AF have shown effects against cerebral edema and increased ICP in models of herpes encephalitis and TBI [8, 12]. Currently, two case-series with reported beneficial effect from Salovum® on ICP in adults with severe TBI have been published [4, 6].

To this date, no medical interventional trials in TBI have succeeded in demonstrating a significant difference in mortality.

In patients were ICP can be controlled, morbidity and mortality is likely to be reduced compared to patients where ICP cannot be controlled [3, 7]. However, the strategies implemented to control ICP could have an impact on morbidity and mortality itself. One of the most efficient ways to control ICP after TBI is by performing a decompressive craniectomy, a procedure that also can influence morbidity and mortality by itself [9]. Due to the fact that Salovum® appears to have no side effects, severe TBI is a diagnosis with high mortality and morbidity without a cure, and promising results from two case-series have been reported, this trial is clinically motivated.

### Objectives

The present trial intends to assess whether Salovum®, an egg yolk powder enriched for AF given to patients with severe traumatic brain injury, will improve outcome as defined by 30-day mortality, ICP, and TIL compared to a control group given placebo egg yolk powder.

## Methods/design

### Trial design

This is a single-center investigator sponsored, phase 2, double-blind, randomized, placebo-controlled, and parallel-arm trial to assess the superiority of AF given as Salovum®, in adult patients with severe traumatic brain injury. Allocation ratio is 1:1. Recruitment commenced in September 2017.

### Trial population and eligibility

A total of 100 adult patients with severe TBI will be enrolled at a single study site, Tygerberg University Hospital, Tygerberg, Cape Town South Africa. Patients with GCS < 9 and indication for invasive ICP monitoring will be screened for inclusion.

### Inclusion criteria

The inclusion criteria are as follows: patients with severe TBI, i.e., Glasgow Coma Score (GCS) < 9 on admission or within 48 h after injury, aged between 18 and 65 years with non-penetrating, isolated head trauma; admission to study hospital within 24 h of injury (for patients with GCS < 9 on admission) and within 24 h of deterioration for patients deteriorating to GCS < 9 within 48 h of injury; no known history of allergy to egg protein; planned for intracranial pressure monitoring and neurointensive care; absence of bilaterally dilated pupils; and CT scan with traumatic pathology that is more than an isolated epidural hematoma.

### Exclusion criteria

The exclusion criteria are as follows: systolic blood pressure below 90 mmHg post resuscitation, epidural hematoma with no other signs of intracranial injury, penetrating injury, and non-fulfillment of inclusion criteria after screening and inclusion.

### Management of traumatic brain injury

The study site will treat all the study patients according to hospital standard of care that may include assisted ventilation, use of invasive ICP monitors, head elevation, hyperventilation, barbiturate coma, mannitol, hypertonic saline, and surgical measures to lower ICP, including decompressive craniectomy.

### Study participants

Study participants will be composed of 100 patients with severe TBI (GCS < 9) that are planned for neurointensive care and an invasive ICP monitor.

### Ethics and protocol

Ethical approval has been granted by the Health Research Ethics Committee (HRECs), Stellenbosch University, Stellenbosch, South Africa (M16/10/040). The study will comply with the ethical principles as set down in the Declaration of Helsinki and will be conducted in accordance with good clinical practice as defined by the International Conference on Harmonization (ICH). The trial is registered with ClinicalTrials.gov, NCT03339505; any amendments to the protocol will be published on ClinicalTrials.gov.

On 14 December 2018, an amendment to the former ethical application was approved by HRECs, stating that recruitment of patients for the trial could be performed with delayed consent for next of kin. The reason for this was that investigators found that relatives of people with no current address were difficult to find within the time frame for inclusion into the trial, thereby creating a potential selection bias.

### Randomization

Patients in the trial are allocated to treatment with Salovum® or placebo egg yolk powder at a ratio of 1:1. Permuted block randomization, with blocks of 4–6, was used and was compiled with Math lab®. Each patient is assigned an envelope, and inside the envelope, the patient study number is written. The patient study number corresponds to a box with the same number, containing the study substance, either active (Salovum®) or placebo (normal egg yolk powder). Study substance and placebo look identical in regard to packaging and the powder itself. Both investigators and patients are blinded during the entire trial. If a single patient needs unblinding, a box with 100 envelopes marked with the patient study number can be accessed. If the envelope with study number is opened, the content will reveal if the patient has received substance or placebo.

### Trial interventions

#### Active therapy

The active therapy is Salovum®, an egg yolk powder enriched for AF, which is manufactured from freeze dried egg yolk (Lantmännen Functional Foods AB, Stockholm, Sweden).

#### Placebo therapy

A placebo powder, containing low amounts of antisecretory factor, made from freeze dried egg yolk and identical in taste, texture, smell, and color to Salovum®, will be used.

#### Dosage

Patients are assigned to 1 out of 3 groups, according to their weight: group 1, < 60 kg, 11 g dose × 6; group 2, 60–80 kg, 14 g dose × 6; and group 3, > 80 kg, 17 g dose × 6. The dosages correspond to doses dispensed in previous studies [4, 6**]**. The study site is equipped with digital scales for weighing the trial substance before administration.

#### Dispensing

Both the Salovum® and placebo substance are packaged in identical bags. After opening of a bag, each containing 20 g, a weighed aliquot depending on patient weight will be mixed with 50–100 ml tap water in a glass container. Electrical milk frothers are used for mixing. The mixture will then be aspired into a syringe and administered to the patient via the nasogastric tubing, used for enteral nutrition.

#### Protocol adherence

Protocol adherence will be encouraged via regular site visits, monitoring, and continuous sponsor-study site communications.

#### Study endpoints

The primary endpoint is the effect of AF, given as a dietary supplement in the form of Salovum®, compared with placebo on mortality at 30 days in adult patients with severe TBI. Comparisons will be made using chi-square/Fisher exact test between active and placebo groups.

Secondary endpoints are ICP and TIL. For ICP, mean values and time over 20 mmHg will be analyzed. Both ICP and TIL will be compared not only between treatment and placebo groups but also between deceased and alive participants within the study groups using non-parametric tests. For comparisons within the groups before and after intervention, the Wilcoxon signed rank test will be used, and for comparisons between the groups, Mann-Whitney *U* test will be used. ICP and TIL will be presented as mean and median values in the respective groups (Table 1).

**Table 1:**
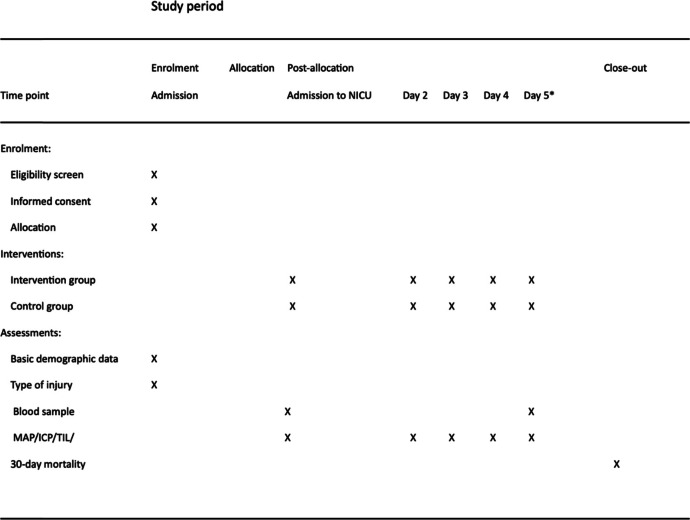
Trial schedule

*Abbreviations*: *NICU* meurointensive care unit, *MAP* mean arterial pressure, *ICP* intracranial pressure, *TIL* treatment intensity level

*Or earlier if substance administration is discontinued before 5 days

#### Data collection of outcome parameters and predefined covariates

All data will be compiled in a paper based CRF and transferred to an electronic CRF continuously. After screening, basic parameters are noted: age, gender, trauma mechanism (motor vehicle accident, falls or blunt trauma), and type of injury (EDH, SDH, contusion, no-mass lesion, SAH).

ICP and mean arterial blood pressure (MAP) will be noted hourly in the patient charts, and TIL will be scored every 24 h. For comparisons between groups, only ICP and TIL during the intervention will be used. For comparisons within groups also, ICP and TIL before intervention can be used.

Once the patient is included in the trial, blood will be drawn and sent for centrifugation of plasma and storage in a − 80 °C freezer. Additionally, a blood sample will be drawn 2–3 days into treatment and after last dosage of trial substance. The plasma will be analyzed for AF levels, markers of brain damage, and cytokines/chemokines for exploratory ad hoc studies.

The total number of days in the neurointensive care unit and days in hospital are noted at the end of the trial for exploratory purposes. The patient or next of kin will be contacted via telephone after 30 days for registration of 30-day mortality. If it is not possible to contact the participants or their next of kin, the population registry will be used. Age, gender, and GCS at inclusion may be used as covariates in exploratory regression analysis.

#### Statistical power of proposed endpoints

The power of the proposed end points was calculated with R statistical software using the Fisher test (1) and power *t*-test (2 and 3) acknowledging the fact that there are no adequate power tests for skewed data.

1. *Mortality*. Reduction of rate of mortality from 40% (20/50 patients) to 16% (8/50 patients) after intervention will give a *p* = 0.01, odds ratio = 0.29, 95% and confidence interval = 0.10–0.80.
2. *Treatment intensity level*. Proposed reduction by 5 grades (delta) after intervention with *n* = 50 in intervention and control group gives power 0.80 with sd = 7.19.
3. *Intracranial pressure (ICP)*. Reduction of ICP after intervention. Proposed reduction by 5 mmHg (delta) or 5 h over 20 mmHg after intervention with *n* = 50 in intervention and control group each gives a power of 0.80 with standard deviation (sd) = 7.19

#### Handling of missing data

With the exception of 30-day mortality, the data collected in this trial is limited to data that is normally measured and registered during standard care at the NICU. Therefore, the amount of missing data is expected to be low. Data missing at random will be handled using last observation carried forward (LOCF). Data not missing at random will be analyzed using mean substitution.

#### Strategies to achieve adequate participant enrolment

The Tygerberg Hospital is tertiary unit with full neurosurgical capacity that serves approximately 3.6 million people and treats approximately 200 patients with TBI and 60 patients with severe TBI each year.

#### Handling of protocol deviations and protocol violations

We defined 4 types of possible protocol deviations/violations in this trial: faulty enrolment of patients, faulty randomization, faulty intervention, and faulty data collection.

The trial is monitored by an external body with intention to disclose any protocol deviations/violations at the end of the trial.

#### Adverse events

As Salovum® is commercially available in Swedish pharmacies and has been available for human use for many years without any reported toxicity, it is not expected to cause any adverse events. Allergy to egg yolk protein (Gal 5; alpha-livetin) has an estimated low incidence in adults (< 0.1%), and anaphylactic reactions are very rare among these. However, special care will be taken to ensure that no vital parameters are changed for the worse in conjunction with the start-up of administering the drug/placebo and at the time for each dose administration, i.e., every 4 h.

The physician responsible for the patient will assess if any adverse or serious adverse events have occurred during the course of each day. The daily patient chart states: “Do you consider that there is a reasonable possibility that an adverse event has been caused by the study compound administered?” The question must be answered daily.

Adverse events: Skin rash and hives

Serious adverse events Serious anaphylactic reaction with hypotension and bronchospasm requiring intervention with corticosteroids and/or vasopressors

#### Data and trial monitoring and interim analysis

An external body, Novotech (see Monitor) monitors the quality of the trail. Three site visits are planned. AE and SAE events related to the trial substance or placebo except a potential egg yolk allergy are not anticipated as no previous side effects have been reported after use of Salovum® in non TBI and TBI patients.

An independent Data and Safety Monitoring Committee (DMC) will perform an unblinded interim analysis when 95 patients have been included. The interim analysis will be conducted by an unblinded statistician and reviewed by the DMC, based on clean data on the primary and secondary outcome variables. The outcome of this interim analysis will result in one of three possible recommendations of the DMC to the sponsor to do one of the following:

- Stop the study because of futility
- Continue and finalize the study as planned
- Continue the study as planned but increase the sample size to a specified number of patients

## Discussion

### Endpoints

Antisecretory factor given as a food supplement, Salovum®, preliminarily appears to have an effect on the, often deleterious, secondary events following severe head trauma [4, 6]. The common view on TBI is that the pathogenic mechanisms are heterogenous and that trials aiming to improve outcome should enroll a large number of participants [17] and use prognostic tools as the IMPACT [19]. This approach could also be based on the fact that no medical intervention has yet changed the outcome of TBI.

Thirty-day mortality, although a crude measure, is robust. The 30-day mortality rate in the placebo group will also give an estimation of the outcome of therapy at the study site for comparison with other sites and settings, as this has not previously been reported. The demographics of the catchment area of the Tygerberg University Hospital makes it difficult to record GOSE at 6 months as part of the outcome. No subgroup or adjusted analyses will be performed. Possibly, attempts to record GOSE or Rankin scores will be made for later ad hoc analysis.

In order to compensate for an increased number of clinical interventions in the arms of the study, the full (summary) treatment intensity level scale (TIL) as opposed to the basic TIL will be used. As the full TIL scale is composed of 8 categories of interventions, it has the capacity to describe virtually all clinical interventions that occur. However, measures, especially decompressive craniectomy (DC), initiated before or just at the beginning of inclusion might obscure the outcome as points for DC will be added for each new 24-h TIL score. Additionally, some patients might not receive the prescribed trial substance for 5 days. Therefore, TIL scoring will be computed for each 24-h period after inclusion. Scores for 24, 48, 72, 96, and 120 h and mean scores divided by time will be used for computations. TIL scoring will begin at the time when the trial substance has been administered for comparisons between the intervention groups, but TIL scores before intervention can be used for comparisons within the intervention groups.

Despite the fact that ICP has been significantly correlated with outcome of TBI in numerous studies, there is no consensus of what level of ICP should be regarded as pathological [3]. With this in mind, analysis of ICP values will be performed addressing both mean changes over the intervention time period between the arms but also within the active arm before and after intervention, when possible. Additional analysis will be conducted comparing the time above proposed normal ICP levels (20 mmHg) for each arm and within the active arm. Further computations of TIL and ICP may be used in exploratory ad hoc studies.

### Dosage

The optimal dosage of Salovum® is not known, nor the optimal interval between doses. However, the substance has been given to a large number of patients with equivalent doses, without any side effects. The dosage used in this trial is also similar to the ones used in reported cases series using Salovum in TBI [4, 6], a pilot trial for treatment of cholera [1] and trials for pediatric diarrhea [24, 25], the latter with a dose up to 16 g to infants 6–24 months old. The rationale behind the dosage and intervals is that previous studies have shown zero toxicity and that the protein is endogenous, which justifies administration of high doses of protein in order to ensure that enough AF-16 is available in the blood to make a clinical difference. AF-16 cannot be accurately measured in blood currently, which makes a dose titration trial futile.

## Trial status

The protocol is version 3, dated November 13, 2020.

This trial is active and has been recruiting since September 22, 2017. Due to the current COVID-19 situation, recruitment has been paused from March to August 2020. Recruitment is estimated to be finalized during 2021.

## Supplementary Information

**Additional file 1.** Full research protocol.

## Data Availability

The full protocol is available as Additional file 1. The full anonymized data set will be available to the public upon reasonable request.
