# Supplementary material for: Effect of antisecretory factor, given as a food supplement to adult patients with severe traumatic brain injury (SASAT): protocol for an exploratory randomized double blind placebo-controlled trial
Source: Trials. 2022 Apr 23;23:340. doi: 10.1186/s13063-022-06275-z (PMC9034076; doi:10.1186/s13063-022-06275-z)
Supplement: Supplementary file 1 — Additional file 1. Full research protocol. [file 13063_2022_6275_MOESM1_ESM.docx]

**Protocol Cover Page**

**Protocol Title:** A prospective, randomized double blind placebo controlled

trial to investigate the effect of *antisecretory factor*,

given as a food supplement to adult patients with severe

traumatic brain injury.

**Protocol Number:** 3

**Protocol Date:** November 13, 2020

**Study Phase:** II

**Project Leader:** Ian Vlok, MD

**Protocol Authors:** David Cederberg, MD

Ian Vlok, MD

Peter Siesjö, MD, PhD, Ass. Prof

**TABLE OF CONTENTS PAGE**

LIST OF ABBREVIATIONS AND DEFINITIONS 3

1. INTRODUCTION 3
   1. Background
   2. Study Rationale
2. STUDY OBJECTIVES 5
3. STUDY PLAN AND PROCEDURES 5
   1. Study Status
   2. Study Design
   3. Study Duration
   4. Study Funding/budget
   5. Study Monitoring
   6. Interim analysis
   7. Study Population
   8. Inclusion Criteria
   9. Exclusion Criteria
   10. Study Enrolment Procedures
       1. Identifying and recruiting candidates for the trial
       2. Consent Procedures
       3. Randomization Procedures
       4. Substance Identity
4. TREATMENT PROTOCOL 7
   1. Patient monitoring – upon arrival at NICU/ICU
   2. General Measures
   3. Respiration
   4. Fluids
   5. Pharmacology
   6. Administration of Salovum® or Placebo
5. STUDY MEASUREMENTS AND ENDPOINTS 10
   1. Medical History/Demographics
   2. Details pertaining the trauma
   3. Physiological and clinical data
   4. Primary endpoints
   5. Secondary endpoints
   6. Monitoring of the trial
6. ADVERSE EVENTS 12
7. STATISTICAL METHODS 12
8. ETHICS 13
   1. Ethics Review
   2. Ethical Conduct of the Trial
   3. Subject Information and Consent
   4. Subject Data Protection
   5. Insurance
9. BUDGET AND SOURCE OF FUNDING 16
10. PUBLICATIONS & PROTOCOL CONTRIBUTIONS 16
11. TRIAL FLOW TABLE 17

12 REFERENCES 16

STUDY CONTACT LIST

Ian Vlok, Head of department

Division of Neurosurgery

Faculty of Medicine & Health Sciences

Stellenbosch University

PO Box 19063 / Francie van Zijl Drive, TYGERBERG, South Africa  7505

**Email:** ianvlok[@sun.ac.za](mailto:mbran@sun.ac.za)   **Tel No.:** +27 (0)21 938 9265   **Fax:** 021 938 5075

Peter Siesjö, Consultant Neurosurgeon, Ass Professor

Department of Neurosurgery

Lund University Hospital

221 85 Lund, Sweden

**Email:** peter.siesjo@med.lu.se   **Tel No.:** +46 705655778 **Fax:** +46 46171817

David Cederberg, Consultant Neurosurgeon

Department of Neurosurgery

Lund University Hospital

221 85 Lund, Sweden

**Email:** david.cederberg@med.lu.se   **Tel No.:** +46 702060344 **Fax:** +46 46171817

**LIST OF ABBREVIATIONS AND DEFINITIONS**

kDa – kilo Dalton - measurement of weight

CSF – Cerebrospinal fluid

AF – Antisecretory factor

ICP – Intracranial pressure

CT – Computerized tomography

GCS – Glasgow Coma Scale – a way of assessing comatose patients

1. **INTRODUCTION**
   1. **Background**

Brain edema arise in trauma and in conjunction with other pathologies in the brain such as infectious diseases, intracranial tumors and ischemic events, i.e. stroke.

Edema can form inside of the cells – intracellular or cytotoxic edema, and outside of the cells – extracellular, vasogenic edema.

The mechanisms underlying the formation of edema are not fully understood. In the injured brain, leakage over the blood brain-barrier arise, transporting fluid from the blood to the extracellular space. This process is disadvantageous because the influx of fluid leads to an increase in distance for diffusion of nutrients and oxygen. The edema increases the intracranial pressure, which leads to a systemic increase in blood pressure, that in turn causes more fluid to leak through the damaged blood brain-barrier. In addition to the physiological, flow-related changes that arise, the edema is also worsened by the inflammatory response to damage. In the injured brain, pro-inflammatory cytokines that can give rise to both intracellular and extracellular edema are released. Also other secondary events such as spreading depression and excitatory damage can worsen the insult.

Existing treatment can, at best, stop the development of edema, and marginally decrease an existing edema. Patients with manifest edema will receive neurointensive care with close monitoring of vital parameters. Patients with a manifest cerebral edema and a decreased level of consciousness are sedated and put on a ventilator. The sedation serves to diminish stress and to lower the basal metabolism of the brain. The lowered metabolism means that the patient´s systemic blood pressure can be kept lower than usual, which in turn means that less blood flows over the damaged blood brain-barrier per time unit, hopefully minimizing the development of further cerebral edema. Sedation and control of the brain´s perfusion pressure is the only available treatment to control and minimize cerebral edema today. There is no specific treatment against the immunological component of cerebral edema.

If the edema becomes expansive enough to give rise to a significant increase in intracranial pressure that may threaten the circulation to the brain surgery must be performed. The most common procedure is hemicraniectomy, which means that the surgeon removes as much as possible of the skull bone over one hemisphere, two hemispheres or bi-frontally. In the procedure, the dura is opened as well. A protective film is placed over the brain and the skin-flap is sutured back in place. After this procedure, the brain may swell out through the bone defects and the intracranial pressure usually normalizes. The procedure itself is risky, and re-operations due to hematoma, CSF-leakage and more are common. Furthermore, it is not known how the procedure itself affects the brain in terms of increased swelling, tearing of the brain etc.

Antisecretory factor (AF) is a 41 kDa protein that exists in most mammals. It was first discovered due to its ability to inhibit experimental diarrhea. AF has been shown to modulate the proliferation of memory/effector T-cells and is expressed by cells in the immune system. In animal experiments, antibodies directed towards AF have been shown to up regulate the pro-inflammatory cytokines IL6 and IL8 and to decrease the anti-inflammatory cytokine IL10. Endogenous AF secretion increases after exposure to bacterial toxins and an increase in AF secretion in combination with an immune reaction may be a part of normal defense against the secretory and inflammatory component in diarrhea. AF has also been shown to be effective against vertigo symptoms in Meniere’s disease, which is caused by an abnormal collection of lymph around the balance nerve. In a trauma model in rats, AF has been proven very efficient in reducing increased intracranial pressure to close to normal values.

The functional amino-terminal part of AF has been synthesized as a 16 amino-acid peptide – AF16. AF16 has been tested in animal experiments. In a herpes encephalitis model in rats, a majority of animals had severely increased intracranial pressure. After administration of AF16, all animals had reduced intracranial pressure. These findings suggest that AF may be involved in modulating the inflammatory processes that lead to cerebral edema and increased intracranial pressure.

AF is commercially available as Salovum®^®^ and is a product based on egg yolk powder B221^®^ as the source for AF. It is available in pharmacies without prescription. Salovum®^®^ is classified as a “medical food” by the European Union (see Appendix 1. Salovum®^®^ is currently registered in the Republic of South Africa.

- 1. **Study Rationale**

To this date, no efficient treatments exist against traumatic cerebral edema. AF has been shown to counteract edema caused by other pathologies in humans. Animal experiments have proven AF to reduce traumatic cerebral edema and reduce development of focal hemorrhagic contusions.

Since 1,5 years, AF has been given to adult patients with severe traumatic brain injury in the Department of Neurosurgery, Lund University Hospital, Sweden. Our preliminary findings suggest that in at least 3 out of 5 patients, AF has had an effect in reducing ICP and thereby avoiding decompressive hemicraniectomy. The study has been granted ethical permission (see Appendix 2). Salovum® has been a classified as a food supplement by the local ethics committee and the Swedish Pharmaceutical Agency (see Appendix 2)

Though, the number of patients with severe traumatic head injury in Sweden is decreasing and the need for a larger, randomized trial in a centre with large volumes of traumatic brain injury is needed

To evaluate if AF can be beneficial for patients in this group, we plan to perform a prospective, randomized double-blinded, placebo-controlled trial.

1. **STUDY OBJECTIVES**

The primary objective of this study is to evaluate the effect of the protein Antisecretory factor (AF), given as a dietary supplement to adult patients with severe traumatic brain injury. We will assess 30-day mortality, ICP, and treatment intensity level (TIL, see Appendix 3) for this purpose.

1. **STUDY PLAN AND PROCEDURES**
   1. **Study status**

After approval by the ethics committee, recruitment started in September 2017. Expected end date is June 2021. The trial was registered at [www.clinicaltrials.gov](http://www.clinicaltrials.gov) with NCT03339505 on September 17, 2017. The study is expected to be completed by June 2021.

- 1. **Study design**

The study is prospective, double-blinded, placebo-controlled, and interventional.

Primary outcome for this study will be 30-day mortality.

Secondary outcomes will be ICP and TIL (Treatment Intensity Level).

Patients that fit the inclusion criteria will be asked to participate via next of kin when admitted to the NICU at Tygerberg University Hospital. If consent is given, the patient will be screened for eligibility. After enrolment in the study, randomization will take place and the patient will be given either Salovum® or egg powder placebo, according to 1g/kg body weight/24 hours. The total dosage will be divided into 6 equal amounts and given to the patient orally via the nasogastric tubing every 4 hours for 5 consecutive days. Placebo group patients will receive supplement according to the same procedure.

ICP, mean arterial pressure (MAP), Treatment Intensity Level (TIL) will be registered for later analysis.

- 1. **Study duration**

The study is anticipated to run for a time period no longer than 4 years.

- 1. **Study funding/budget**

1100 000 Rands for covering costs for the trial will be provided from research funds at Lund University Hospital, Sweden.

- 1. **Study monitoring**

The study will be monitored by an external monitoring company. Monitoring will take place at regular intervals (2-4 times per year, depending on recruitment progression) Novotech (novotech-cro.com, Ground Floor, Building 4, Quadrum Office Park, 50 Constantia Boulevard, Constantia Kloof Johannesburg Gauteng 1709 South Africa).

- 1. **Interim analysis**

An independent Data and Safety Monitoring Committee (DMC) will perform an unblinded interim analysis when 95 patients have been included. The interim analysis will be conducted by an unblinded statistician and reviewed by the DMC, based on clean data on the primary

and secondary outcome variables. The outcome of this interim analysis will result in one of three possible recommendations of the DMC to the Sponsor to do one of the following:

- Stop the study because of futility
- Continue and finalize the study as planned
- Continue the study as planned but increase the sample size to a specified number of patients
  1. **Study population**

The study population will be adult patients (18-65 years) that are treated in the NICU for severe traumatic brain injury. Recruitment will be via the patients´ next of kin, who will be asked about participation in the study. Recruitment will continue until 100 patients have been randomized into the 2 groups (active substance and placebo) of 50 patients each. If no next of kin can be found, the patient can be included in the study according to paragraph 30 Helsinki Declaration (see also 3.7.2).

- 1. **Inclusion criteria**

To be enrolled, the following criteria have to be fulfilled:

1. Adult of either gender between 18 and 65 years.
2. Non-penetrating, isolated severe traumatic brain injury
3. GCS >3 and GCS<9 on admission or within 48 hours after injury*
4. Admission to study hospital within 24 hours of injury*
5. No known history of allergy to egg-protein
6. Planned for intracranial pressure monitoring
7. Absence of bilaterally dilated pupils
8. CT scan with traumatic pathology that is more than an isolated epidural hematoma

*Within 24 hours of injury (for patients with GCS < 9 on admission) or

Within 24 hours of deterioration (among patients deteriorating to GCS < 9 within 48 hours of injury)

- 1. **Exclusion criteria**

Any of the following is regarded as a criterion for exclusion from the study:

1. No consent
2. Systolic blood pressure below 90 mm Hg post resuscitation
3. Non-fulfillment of inclusion criteria after screening and inclusion procedures.

**Study enrolment procedures**

- - 1. **Identifying and recruiting candidates for the trial**

When a patient with severe traumatic brain injury is admitted to the Neurointensive Care Unit, the neurosurgeon on call will evaluate the patient, ask for informed consent from next of kin, if available, and use the screening protocol for inclusion.

All patients that are screened will be recorded in the screening log. Reasons for ineligibility and for non-participation of eligible candidates will be recorded in the log.

- - 1. **Consent procedures**

The study coordinator or a person authorized by the study coordinator will come to the bedside of the patient and introduce him/her-self to the relatives. The project will be described in general terms and the relatives of the patient will be informed that the patient is eligible for inclusion in the study. The study coordinator will take the family to a place where they can talk confidentially, and describe every aspect of the project. Time will be given to ask questions, and the family will be asked to repeat back in their own words what has been discussed, to make sure that they understand. The potential advantages and disadvantages of participating in the study will be described, and the care of the patient if included in the study, and if not in the study will be described.

The family will be especially informed that participation in the study is completely voluntary and that if they wish to participate, they are free to quit the study at any time.

Next to kin of the eligible participant will be provided with written information about the study, which also includes complete contact information for the study coordinator and co-investigators. Written approval will be obtained in the presence of a witness.

The Investigator will store the original version of the Subject Information and Consent Form and a copy will be given to the subject´s family. Samples of the English version of the Subject Information and Consent Forms will be enclosed. Afrikaans and Xhosa translations of the approved English version will also be provided and enclosed in the Ethics application.

If next of kin is not found after screening of a patient the patient can be included in the study according to the latest Helsinki Declaration (2013) , paragraphs 28-30 where it is stated that if a patient is incapable of giving consent due to a condition that is a necessary characteristic of the research group (eg severe TBI) , the intervention cannot be delayed, there is likelihood that the intervention will benefit the participant and there are minimal risk with the intervention. If a patient s included without consent from next of kin, consent to remain in the research must be obtained as soon as possible from next of kin.

- - 1. **Randomization procedure**

Patients will be assigned a number between 1 and 100. 50 boxes containing Salovum®^®^ and 50 boxes containing ordinary egg yolk powder will be assigned a number between 1 and 100. The study will be conducted in a double-blind fashion and neither the investigators nor the patients will know who receives Salovum®^®^.

- - 1. **Substance identity**

Salovum®^®^ will be packaged into 5 plastic bags each containing 100g which will be packaged into one bag. 50 bags of Salovum®^®^ will be sealed and marked with a random number between 1 and 100.

Identically looking placebo, ordinary egg yolk powder will be used for placebo group. Placebo will be packed the same way as Salovum®^®^ and 50 packages will be marked with random numbers between 1 and 100.

The key to which boxes that contain active substance will be kept in two sealed envelopes that will be kept locked up until after completion of the study. Individual patients may be unblinded in the case of a serious adverse event. A locked box at the study site, containing 100 sealed envelopes can be accessed in the case of a serious adverse event. The investigator can open the envelope containing the patients´ study number and inside the envelope it is clearly stated if the patient has received Salovum® or placebo.

**TREATMENT PROTOCOL**

- 1. **Patient monitoring – upon arrival at NICU/ICU**
- Place continuous SaPO2 monitor
- Insert indwelling urinary catheter to monitor urine output
- Insert gastric tube to avoid gastric retention, and for administration of Salovum® and placebo.
- Insert arterial catheter for arterial mean pressure monitoring – MAP should be zeroed at the level of Foramen Monroe.
- Monitor pupil size and reactivity to light each hour
- New CT scan if clinically motivated
- Insert intracranial pressure monitoring device – intra-parenchymal or intraventricular. Intraventricular monitors should be zeroed at the level of the foramen of Monroe.
  1. **General measures**
- Head and neck in neutral position and aligned
- Avoid hyperthermia - Defined as central temperature ≥ 38 ° C
  - - - - Paracetamol
- Early enteral nutritional support
  - - - - Before 48 hours
        - 15-20 Kcal/kg weight
- Pharmacologic prophylaxis if post traumatic seizures
  - - - - Drug according to local praxis
- Gastric bleeding prophylaxis
  - - - - Drug according to local praxis
- Avoid deep venous thrombosis
  - - - - Proceed according to local praxis
- Frequent tracheal suctioning with sterile technique to prevent pulmonary infections
  1. **Respiration**
- Mechanical ventilation.
  - - - - Ventilate patient to normal PaCO2 (4,6 – 5,2 kPa), normal PaO2 (12-14 kPa) PEEP ≈ 5 cm H2O, and intermittent bagging to prevent atelectasis.
- Hyperventilation – PaCO2 between 4,0 – 4,5, only as a temporary measure to cope with an ICP-crisis according to local practice.
  1. **Fluids**
- According to local praxis
  1. **Pharmacology**
- According to local praxis
- Use of Pentothal will be noted in protocol
  1. **Administration of Salovum® or Placebo**
- All patients enrolled in protocol must have a gastric feeding tube.
- Administration of Salovum® or placebo - 1 g per patient weight in kg per 24 hours divided into 6 administrations, dissolved in 50-100 ml of water per administration.
- Treatment will be given for 5 days/120 hours = 30 administrations, or until the ICP monitor is removed.

1. **STUDY MEASUREMENTS AND ENDPOINTS**

In this study the following data will be measured/recorded in a patient specific folder, clearly marked with the patient study number. Data will later be transferred to an excel sheet for statistical analysis. The excel sheet will only contain study number. Data that can be used to identify the study participants will be stored in a locked cabinet in the study site.

- 1. **Medical history/demographics**

The occurrence of diabetes and antithrombotic therapy prior to present trauma will be noted in addition to MRN, Date of birth, gender and address.

- 1. **Details pertaining the trauma**

Trauma mechanism will be noted. Time elapsed from trauma to assessment by neurosurgeon. Pupil status will be recorded at screening, when assessed by neurosurgeon.

- 1. **Physiological and clinical data**

Patient ICP and Mean Arterial Blood Pressure (MAP) will be recorded at screening/inclusion and on an hourly basis.

Patient GCS will be recorded at screening/inclusion. Neurointensive care specific treatment will be registered as TIL (Treatment Intensity Level) every 24 hours. The TIL scale used will be the differentiated TIL scale with a score ranging from 0-38 (Appendix 3).

All clinical data will be recorded for as long as the patient is treated in the neurosurgical ICU.

Gastro paresis will be evaluated every 4 hours by aspirating via the patients´ nasogastric tube, prior to administration of Salovum®/Placebo. Gastro paresis will be evaluated during the entire treatment time (see 4.6)

- 1. **Blood samples**

Once the patient is included in the trial, blood will be drawn and sent for centrifugation of plasma and storage in a -80°C freezer. Additionally, a blood sample will be drawn 2-3 days into treatment and after last dosage of trial substance. The plasma will be analyzed for AF levels, markers of brain damage and cytokines/chemokines for exploratory ad-hoc studies.

- 1. **Primary end point**

Mortality at 30 days

- 1. **Secondary end points**

TIL – Treatment Intensity Level

Intracranial pressure (ICP)

1. **ADVERSE EVENTS**

An adverse event (AE) is the development of disadvantageous medical symptoms or conditions, or deterioration of a pre-existing medical condition, that occur as the result of participating in the study.

As Salovum® is commercially available in Swedish pharmacies and has been available for human use for many years without any reported toxicity, it is not expected to cause any adverse events. Allergy to egg yolk protein (Gal 5; alpha-livetin) has an estimated low incidence in adults (< 0.1 %) and anaphylactic reactions are very rare among these. However, special care will be taken to ensure that no vital parameters are changed for the worse in conjunction with the start-up of administering the drug/placebo, and at the time for each dose administration, i.e. every 4 hours.

The physician responsible for the patient will assess if any Adverse or Serious Adverse Events have occurred during the course of each day. The daily patient chart states: “Do you consider that there is a reasonable possibility that an adverse event has been caused by the study compound administered?” The question must be answered daily.

Adverse events:

Skin rash and hives.

Serious adverse events

Serious anaphylactic reaction with hypotension and bronchospasm requiring intervention with corticosteroids and/or vasopressors.

1. **STATISTICAL METHODS**

**Mortality at 30 days**

The power of the proposed end points was calculated with R statistical software using the Fisher test (1) and power t-test (2 and 3) acknowledging the fact that there are no adequate power tests for skewed data.

*Mortality*. Reduction of rate of mortality from 40% (20/50 patients) to 16% (8/50 patients) after intervention will give a p=0.01, odds ratio=0.29, 95% confidence interval=0.10-0.80.

*Treatment intensity level*. Proposed reduction by 5 grades (delta) after intervention with n=50 in intervention and control group gives power 0.80 with sd=7.19.

*Intracranial pressure (ICP)*. Reduction of ICP after intervention. Proposed reduction by 5 mm Hg (delta) or 5 hours over 20 mm Hg after intervention with n=50 in intervention and control group each gives a power of 0.80 with standard deviation (sd)=7.19

**AE and SAE**

These parameters will be reported and handled according to the ethical permit and GCP

1. **ETHICS**
   1. **Ethics review**

Ethical approval has been granted by the Health Research Ethics Committee (HRECs), Stellenbosch University, Stellenbosch, South Africa (M16/10/040). The study will comply with the ethical principles as set down in the Declaration of Helsinki, and will be conducted in accordance with good clinical practice as defined by the International Conference on Harmonization (ICH). The trial is registered with ClinicalTrials.gov, NCT03339505.

On 14/12/2018, an amendment to the former ethical application was approved by HRECs, stating that recruitment of patients for the trial could be performed with delayed consent for next of kin. The reason for this was that investigators found that relatives of people with no current address were difficult to find within the time frame for inclusion into the trial, thereby creating a potential selection bias.

- 1. **Ethical conduct of the trial**

The study will be performed in accordance with the ethical principles in the Declaration of Helsinki, and that are consistent with Good Clinical Practice and other applicable regulatory requirements.

**8.2.1** **Randomization**

Both the investigator and the patient will be blinded as to the content of the egg powder administered. The randomization will be concealed by means sealed envelopes clearly indicating the randomization once fulfilled. The randomization procedure will yield a unique number and the package of egg powder with the corresponding number administered to the randomized participant.

**8.2.2** **Beneficence**

Those randomized to the treatment arm might benefit from the study. As no specific treatment against traumatic brain edema exists today, any effect Salovum® might have on decreasing the edema, will likely benefit patients in the treatment arm. Apart from having a possible effect on cerebral edema and subsequently intracranial pressure, Salovum® could also have an indirect beneficial effect by reducing both surgical and pharmacological measures, otherwise taken to lower intracranial pressure. The study might also be of great benefit to future patients with traumatic brain injury, since the information gained from the study can be used to evaluate the role of Salovum® in upcoming trials of traumatic brain injury.

**8.2.3** **Non-maleficence**

The risk of harm to participants is estimated to be extremely low. The management of severe traumatic brain injury for all participants in the study conforms to the current protocols and practices at Tygerberg Hospital, and will be identical between the two groups. There will also be no deviation in the surgical and follow-up regimes of the patients involved. The only difference between the two groups will be that one group will receive Salovum® (e.g. antisecretory factor enriched egg yolk powder) as part of their daily dietary intake, and the other group will receive ordinary egg yolk powder. Both supplements are high in protein and contain only natural nutrients that will be part of the patients´ daily nutritional demand. Salovum® has been used for many years in high doses for patients with various diagnoses, and there is no known toxicity, neither has there been any signs of immune reactivity in the form of antibody production. Salovum® has been given to adult patients with severe traumatic head injury in the department of Neurosurgery, Lund University Hospital, Lund, Sweden, during a clinical trial since January 2014. Salovum® has been shown to have a very promising effect on lowering ICP and decreasing the need for re-operations in this trial without any signs of toxicity.

**8.2.4 Justice**

All participants will be included in the study if they meet the inclusion and exclusion criteria as stated in the protocol. No person will be included or excluded based on any personal characteristics.

**8.2.5 Autonomy**

Participation in the study is entirely voluntary. Even if participants have opted to participate, they could withdraw from the study at any stage and such a decision will not have any impact on their further management and medical or surgical treatment.

**8.2.6** **Informed consent**

Written informed consent will be requested for all study participants via next of kin. If no next of kin can be found, the patient may be included in the study without informed consent (see 8.1) The study aim and objectives, as well as the procedures will be explained to the individual by the investigators and they will be given sufficient opportunity to ask questions. Each participant will be provided with a copy of the informed consent document, which would include important contact details. Informed consent will be managed as an ongoing process and the information will be re-enforced with each visit.

**8.2.7** **Independent review**

The study will adhere to the principles enshrined in the Declaration of Helsinki as well as South African Good Clinical Practice guidelines, standards and legislation. The protocol will be submitted for independent review by the Health Research Ethics Committee, University of Stellenbosch.

**8.2.8** **Confidentiality**

All investigators involved in the study are part of the clinical team managing these patients. No non-medical person will have access to the information. All names and other identifiable numbers and data items will be removed when the data set is locked for analysis. Each participant will be provided with a unique study number, which will be used for all documentation. The unique study numbers will be linked to the identifiable data in a separate database, which will be password protected and kept on the personal computer (also password protected) of the primary investigator.

**8.2.9** **Conflicts of interest and sources of funding**

There will be no patient remuneration for participation in this study. None of the investigators or sponsors will benefit financially from the conduct of this trial. All analyzes, interpretations and publications of data will be performed independently by the investigators. The investigators have no other competing interests or conflicts of interest to declare. Funding to cover costs for screening, inclusion, treatment and follow-up will be supplied by research funds from the Department of Neurosurgery, Lund University Hospital, Sweden.

### 8.2.10 Insurance

Standard Hospital Insurance will cover all subjects participating in the trial.

## 9 Budget and source of funding

| **Post** | **Cost (R)** | **Source** |
| --- | --- | --- |
| Administrative costs, ethical application, translations etc. | 16000 | Lund University Hospital |
| Upgrade of system for central data collection in NICU | 484000 | Lund University Hospital |
| Compensation to cover extra expenses 5000 R/patient | 500000 | Lund University Hospital |
| Extra to cover unanticipated costs | 100000 | Lund University Hospital |
| **Summary** | **1100000** | Lund University Hospital |

## 10 Publications & Contributions

All results from this study will be admitted for publication in a peer-reviewed scientific journal. Publications will be admitted as a joint collaboration between Stellenbosch and Lund University. DC contributed to the design of the trial and the statistical analysis plan, drafted the manuscript and is sponsor/investigator. BH is investigator at the trial site and reviewed the manuscript. AV is the principal investigator at the trial site and reviewed the Manuscript. PS is principal sponsor /investigator, contributed to the trial design, reviewed the manuscript and drafted the statistical analysis plan. All authors read and approved the final manuscript.

**11 Trial flow table**


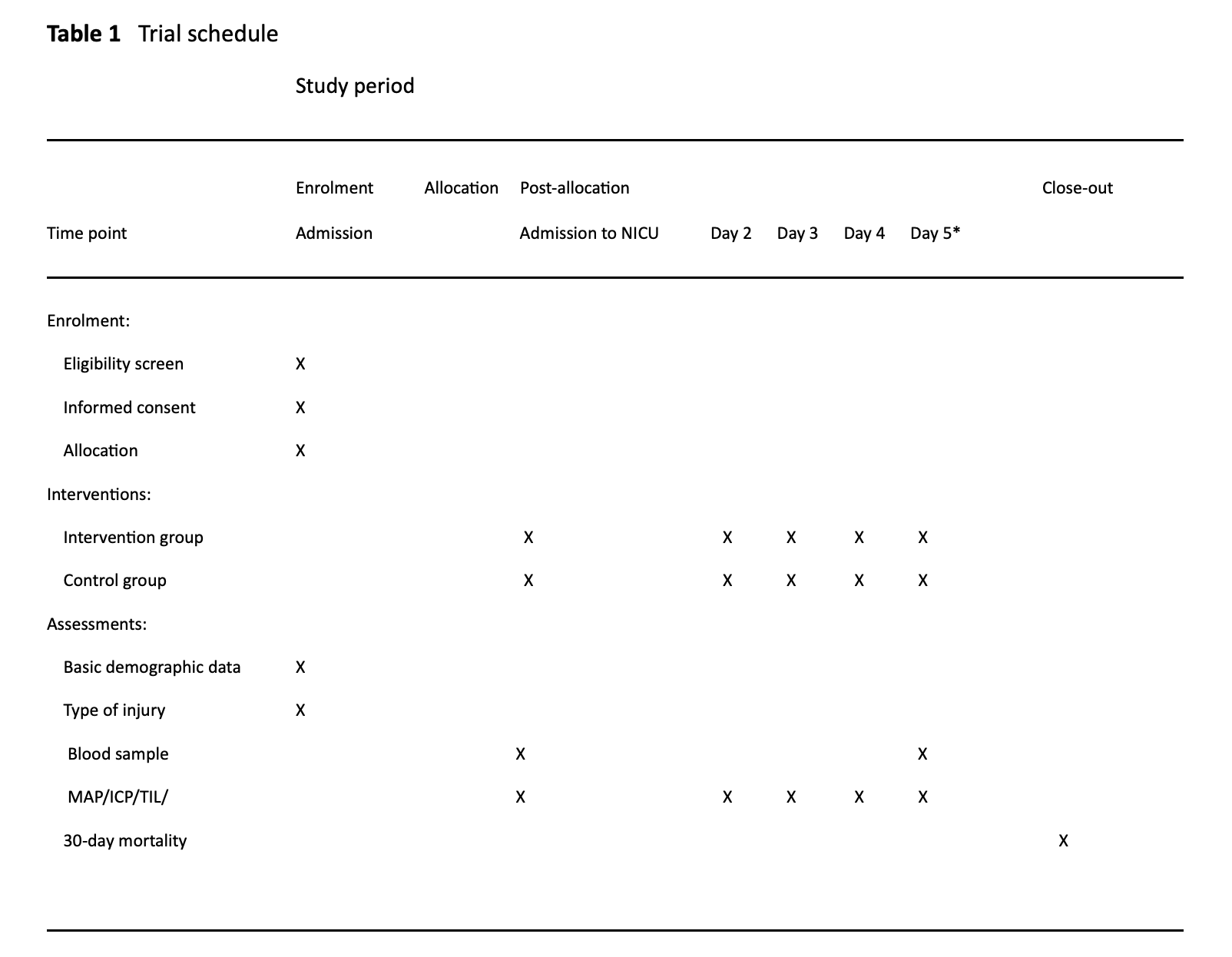


## 12 References

1. Abbott, N. J., Patabendige, A. A. K., Dolman, D. E. M., Yusof, S. R., & Begley, D. J. (2010). Structure and function of the blood-brain barrier. *Neurobiology of Disease*, *37*(1), 13–25. doi:10.1016/j.nbd.2009.07.030
2. Al-Olama, M., Wallgren, A., Andersson, B., Gatzinsky, K., Hultborn, R., Karlsson-Parra, A., et al. (2011). The peptide AF-16 decreases high interstitial fluid pressure in solid tumors. *Acta Oncologica (Stockholm, Sweden)*, *50*(7), 1098–1104. doi:10.3109/0284186X.2011.562240
3. Armulik, A., Genové, G., Mäe, M., Nisancioglu, M. H., Wallgard, E., Niaudet, C., et al. (2010). Pericytes regulate the blood-brain barrier. *Nature*, *468*(7323), 557–561. doi:10.1038/nature09522
4. Davidson, T. S., Davidson, T. S., Hickey, W. F., & Hickey, W. F. (2004a). Antisecretory factor expression is regulated by inflammatory mediators and influences the severity of experimental autoimmune encephalomyelitis. *Journal of Leukocyte Biology*, *76*(4), 835–844. doi:10.1189/jlb.0204085
5. Davidson, T. S., Davidson, T. S., Hickey, W. F., & Hickey, W. F. (2004b). Distribution and immunoregulatory properties of antisecretory factor. *Laboratory Investigation; a Journal of Technical Methods and Pathology*, *84*(3), 307–319. doi:10.1038/labinvest.3700036
6. Hanner, P., Rask-Andersen, H., Lange, S., & Jennische, E. (2010). Antisecretory factor-inducing therapy improves the clinical outcome in patients with Ménière's disease. *Acta Oto-Laryngologica*, *130*(2), 223–227. doi:10.3109/00016480903022842
7. Hansson, H.-A., Al-Olama, M., Jennische, E., Gatzinsky, K., & Lange, S. (2012). The peptide AF-16 and the AF protein counteract intracranial hypertension. *Acta Neurochirurgica Supplement*, *114*, 377–382. doi:10.1007/978-3-7091-0956-4_73
8. Jennische, E., Bergström, T., Johansson, M., Nyström, K., Tarkowski, A., Hansson, H.-A., & Lange, S. (2008). The peptide AF-16 abolishes sickness and death at experimental encephalitis by reducing increase of intracranial pressure. *Brain Research*, *1227*, 189–197. doi:10.1016/j.brainres.2008.05.083
9. Johansson, E., Al-Olama, M., Hansson, H.-A., Lange, S., & Jennische, E. (2013). Diet-induced antisecretory factor prevents intracranial hypertension in a dosage-dependent manner. *The British Journal of Nutrition*, *109*(12), 2247–2252. doi:10.1017/S0007114512004552
10. Nag, S., Kapadia, A., & Stewart, D. J. (2011). Review: molecular pathogenesis of blood-brain barrier breakdown in acute brain injury. *Neuropathology and Applied Neurobiology*, *37*(1), 3–23. doi:10.1111/j.1365-2990.2010.01138.x
11. Nicolas V, Liévin-Le Moal V. Antisecretory factor peptide AF-16 inhibits the secreted autotransporter toxin-stimulated transcellular and paracellular passages of fluid in cultured human enterocyte-like cells. Infect Immun. 2015 Mar;83(3):907–22.
12. Matson Dzebo M, Reymer A, Fant K, Lincoln P, Nordén B, Rocha S. Enhanced cellular uptake of antisecretory peptide AF-16 through proteoglycan binding. Biochemistry. 2014 Oct 21;53(41):6566–73.
13. Eide PK, Eidsvaag VA, Hansson H-A. Antisecretory factor (AF) exerts no effects on intracranial pressure (ICP) waves and ICP in patients with idiopathic normal pressure hydrocephalus and idiopathic intracranial hypertension. J Neurol Sci. 2014 Aug 15;343(1-2):132–7.
14. Zaman S, Aamir K, Lange S, Jennische E, Silfverdal S-A, Hanson LÅ. Antisecretory factor effectively and safely stops childhood diarrhoea: a placebo-controlled, randomised study. Acta Paediatr. 2014 Jun;103(6):659–64.
15. Leong SC, Narayan S, Lesser TH. Antisecretory factor-inducing therapy improves patient-reported functional levels in Meniere's disease. Ann Otol Rhinol Laryngol. 2013 Oct;122(10):619–24.
16. Mañé J, Lorén V, Pedrosa E, Ojanguren I, Domènech E, Gassull MA, et al. Therapeutic effect of antisecretory factor-rich egg yolk on the late phases of 2,4,6-trinitrobenzenesulphonic acid colitis in mice. Br J Nutr. 2011 Nov;106(10):1522–8.
17. Alam NH, Ashraf H, Olesen M, Salam MA, Gyr N, Meier R. Salovum® egg yolk containing antisecretory factor as an adjunct therapy in severe cholera in adult males: a pilot study. J Health Popul Nutr. 2011 Aug;29(4):297–302.
18. Tateishi K, Misumi Y, Ikehara Y, Miyasaka K, Funakoshi A. Molecular cloning and expression of rat antisecretory factor and its intracellular localization. Biochem Cell Biol. 1999;77(3):223–8.
19. Zuercher P, Groen Justus L, Aries Marcel J.H. Reliabiltiy and Validity of the Therapy Inensity Level Scale: Analysis of Clinimetric Properties of a Novel Approach to Assess Management of Intracranial Pressure in Traumatic Brain Injury. Journal of Neurotrauma. 2016 Oct; 33:1768-1774
20. Urbaniak, G. C., & Plous, S. (2013). Research Randomizer (Version 4.0) [Computer software]. Retreived on June 22, 2013, from <http://www.randomizer.org/>
